# Electrical signalling and plant response to herbivory: A short review

**DOI:** 10.1080/15592324.2023.2277578

**Published:** 2023-12-05

**Authors:** Jéssica K.S Pachú, Francynes C.O. Macedo, José B Malaquias, Francisco S. Ramalho, Ricardo F. Oliveira, Wesley A.C Godoy, Angélica S. Salustino

**Affiliations:** a Department of Entomology and Acarology, Luiz de Queiroz College of Agriculture (ESALQ), University of São Paulo (USP), Piracicaba, São Paulo, Brazil; b Department of Biological Sciences, Luiz de Queiroz College of Agriculture (ESALQ), University of São Paulo (USP), Piracicaba, São Paulo, Brazil; c Entomology Laboratory, Agrarian Science Center, Federal University of Paraíba, Areia, Brazil; d Biological Control Unit, Empresa Brasileira de Pesquisa Agropecuaria, Campina Grande, Brazil

**Keywords:** electrical signals, stress response, herbivory

## Abstract

For a long time, electrical signaling was neglected at the expense of signaling studies in plants being concentrated with chemical and hydraulic signals. Studies conducted in recent years have revealed that plants are capable of emitting, processing, and transmitting bioelectrical signals to regulate a wide variety of physiological functions. Many important biological and physiological phenomena are accompanied by these cellular electrical manifestations, which supports the hypothesis about the importance of bioelectricity as a fundamental ‘model’ for response the stresses environmental and for activities regeneration of these organisms. Electrical signals have also been characterized and discriminated against in genetically modified plants under stress mediated by sucking insects and/or by the application of systemic insecticides. Such results can guide future studies that aim to elucidate the factors involved in the processes of resistance to stress and plant defense, thus aiding in the development of successful strategies in integrated pest management. Therefore, this mini review includes the results of studies aimed at electrical signaling in response to biotic stress. We also demonstrated how the generation and propagation of electrical signals takes place and included a description of how these electrical potentials are measured.

The ability to respond to environmental stimuli is a basic ability necessary for an organism to live and thrive. When dealing with sessile beings, such as plants, this ability is even more crucial considering that the environment changes constantly. Plants need to respond quickly to these changes, as under certain conditions, they can cause reversible and/or irreversible damage to the plant. Understanding how plants perceive and react to the most diverse environmental stimuli, especially harmful stimuli, has been the object of study by many researchers around the world.^1^ The main justification for this interest is that environmental changes can cause biotic and abiotic damage or stress, limiting plant growth and development. Therefore, understanding how plants behave under stress conditions is of great importance and involves the effort of several areas of plant sciences, such as biochemistry, physiology, genetics, molecular biology, ecology, and electrophysiology.^2^

The change electrical signaling as response of the plant to herbivory is a universal and fast reaction to external stimuli.^3^ Among the various biotic stress factors, herbivory is one of the most prevalent, affecting plant lives. Herbivory is caused by mammals or phytophagous insects, and in addition to damaging plant tissue, it also opens “doors” for the attack of pathogenic microorganisms that cause disease, which in turn characterizes another stress factor. The plant-herbivore interaction, although widely studied, is very complex and still contains many obscure aspects. However, plants are not passive organisms and have several defense strategies against herbivory, including the presence of mechanical barriers, such as thorns, and chemical barriers, such as the production of alkaloids, terpenoids, steroids, phenolic compounds, and other secondary metabolites that can be toxic to animals, have an unpleasant taste, or even serve as a signal to warn neighboring plants about the attack.^4^ For stress responses to be effective, plants need to perceive the stress and then activate mechanisms that lead to a local or systemic defense response; that is, there needs to be a signal to link perception and response. A key factor in these signaling mechanisms is jasmonic acid, a lipid-derived plant hormone that rapidly accumulates in organs that are remote from the herbivore’s feeding site. Electrical signals also act in this signaling process,^5,6^ being able these increase the production of jasmonic acid in plant leaves.^7^

Bioelectrical activity due to stimulation in plants was first discovered by Burdon-Sanderson in 1873. He measured an action potential that propagated at a speed of 200 mm s-1 in *Dionaea muscipula* and occurred under the strong influence of temperature. Sanderson also observed that action potential propagation occurs in the central portion of the leaf blade, being faster in the abaxial face, and is directed perpendicularly to the midrib.^8^ Later, electrical signals in plant cells were discovered and studied in various plants.

At the resting membrane potential, living cells present an electrical potential difference of several tens of millivolts across the plasma membrane, with the intracellular medium negative in relation to extracellular fluids. The genesis of this membrane potential is associated with ion transport mechanisms, which create an intracellular ionic medium with a different composition of the extracellular ionic medium. Therefore, diffusion processes and active transport represent the basic mechanisms responsible for the polarization of the plasma membrane.

The action potential (AP) is a signal propagating transient depolarization with a characteristic impulse form; they possess amplitudes from several tens to one hundred millivolts (mV) and durations from several seconds, in locomotive plants, to several tens of seconds in ordinary plants. Variation potential (VP), otherwise termed as “slow wave potential”, is a transient depolarization of an irregular shape, with an amplitude of several tens of mV and a duration of up to several tens of minutes.^9^ Intracellular electrical signals serve as a mode of information transmission in plant cells.^10^ Long-distance electrical signaling is involved in several physiological processes, such as photosynthesis,^11^ cell elongation (Shiina & Tazawa, 1986),^49^ respiration,^12^ water absorption,^13^ gas exchange,^14^ phloem transport,^15^ pollination,^16^ and many other vital processes.^17^ Furthermore, studies have shown that plants have greater electrical excitability under unfavorable conditions, which is due to the need to respond quickly to environmental stress caused by both biotic^18^ (Pachú et al., 2021)^50^ and abiotic factors.^19^ In this brief review, we addressed general aspects of plant electrophysiology, such as the types of electrical signals and methods for recording the electrical activity of plants, and highlighted the role of electrical signals in plant responses to biotic stress.

## Types of electrical signals in plants

The main electrical signals in plants are the AP, the variation potential (VP), and the system potential (SP). The AP transmits at a constant speed and maintains a constant amplitude; it follows the all-or-nothing law, in which stimuli weaker than a certain threshold can generate a change in this potential. After AP is generated, the cell membrane enters absolute and relatively refractory periods in succession.^20^ Action potentials are induced by nonharmful stimuli, e.g., cold, mechanical, and electrical stimuli (Opritov et al., 2002;^52^ Krol et al., 2003;^21,22,^^53^ and are a signaling phenomenon that can transmit information quickly over long distances. APs are based on the activity of voltage channels, with calcium, chlorine, and potassium being the main ions involved in the mechanisms of generation of this signal in plants.^23,24^

The VP, also called slow wave potentials, are not constant and decrease in speed and amplitude as they move away from the stimulus site. They are induced by harmful stimuli (e.g., burning and cutting;^10^ and are characterized by long-term depolarization whose duration can be tens of minutes or longer.^20,25^ VP generation occurs after activation of mechanosensitive or ligand-dependent Ca^2+^ channels induced by hydraulic waves or wound substance propagation,^26^ which leads to increased intracellular calcium concentrations and consequent inactivation of H^+^ - ATPase in the plasma membrane.^27^ SP is a systemic signal induced by abiotic and biotic factors that is self-propagating and transmitted via a hyperpolarisation event related to the activation of H^+^-ATPase in the plasma membrane.^28^ However, the participation of Ca^2+^ and K^+^ channels in SP propagation is likely because this signal was suppressed in plants deficient in these nutrients.^29^ ZIMMERMANN et al.^30^ also observed the propagation of SP in the stimulation zone after the induction of VP and/or AP, indicating that both depolarization and hyperpolarisation in the stimulated zone must induce some similar processes that participate in the propagation of SP.^17^

## Methods for recording electrical activity in plants

Approaches to the study of electrical activities in plants include intra- and extracellular measurements. Extracellular measurement is a noninvasive and physically stable technique, and measurements may be performed simultaneously with other physiological methods, such as gas exchange and plant turgor; this also applies to tests to observe the variation in electrical potential in the long term (>24 h); ;.^31^ Measurements are made using electrodes that consist of an Ag/AgCl lead wire moistened with 0.1% KCl (w/v) in agar and wrapped in cotton to promote proper contact with the plant surface;^32^ alternatively, the electrodes can be connected to the plant surface using a conductive aqueous gel, which is commonly used in electrocardiography (Mancuso, 1999).^51^ Other recent and advanced methods have been used to quantify electrical activity in plants, Parise et al.,^33^ for example, found that there is a pattern recognizable by machine learning techniques in the electrome of the dodders presented to different hosts.

A four-channel data acquisition interface and software (Lab-Trax 4/24T, World Precision Instruments and LabScribe® version 3, iWorx Systems Inc.) are required. Each channel is independent, with its own 24-bit analogue-to-digital converter and equipped with the appropriate filters. The electrical signals in plants need to be amplified, and the recording device must have a high input impedance. The electrodes can be connected by cables to a computer screen, and an identical electrode must be connected in the distal region of the plant or to the ground to serve as a reference electrode. When the various channels show stabilized potentials, the plant can be electrically stimulated (3 V for 2 s) or by another stimulus (heat, cold, cut) applied to the leaf. Signals from the electrodes are then amplified and recorded, and usually, the electrical response can be verified in all electrodes, indicating that the transmission of the electrical signal occurs throughout the plant.^10,31^

The intracellular measurement technique applies to the observation and study of bioelectricity at the cell level and typically uses a glass microelectrode with a tip diameter of less than 1 μm inserted into the cell. These are very accurate measurements but punctual because, as this is an invasive technique, the measurement is done in a short time because the electrolytes present in the electrode can diffuse into the cell, changing the original bioelectrical conditions.^10,34^ The microelectrodes are filled with KCl, fixed to an Ag/AgCl wire, and connected to an amplifier. After the amplifier has been reset with both electrodes outside the cell, a microelectrode is inserted into the cytoplasm (or vacuole) of a cell with a micromanipulator, and the reference electrode is placed in the solution around the cell.^10^

## Electrical signalling in response to biotic stress

Different environmental stimuli evoke specific responses in living cells, which are rapidly transmitted over long distances.^29^ For example, the propagation of electrical signals that act in the rapid formation of adaptive responses generated by plants after the action of their stressors, increasing their tolerance^7^. Given the importance of electrical signals numerous physiological effects of electrical signaling have been described in recent years;^20^ .^35–37^

Electrical signals have been elucidated as one of the main responses to herbivory, occurring within seconds to minutes after the injury occurs and followed by a cascade of chemical signals.^38,39^ Volkov and Haack (1995)^54^ were the first to measure insect-induced action and variation potentials in long-distance plant communication. The experiment was carried out with potato plants (*Solanum tuberosum* L.) In the presence of Colorado potato beetle larvae (*Leptinotarsa decemlineata* (Say); Coleoptera: Chrysomelidae), the larvae consumed the upper leaves of the plants, and after 6–10 h, action potentials with amplitudes of 40 mV were recorded every 2 0.5 h during a 2-day test period. In studies with *Spodoptera littoralis* (Boisduval) (Lepidoptera: Noctuidae) in *Phaseolus lunatus*, both direct herbivory and oral secretions of the insect induced a rapid depolarization of Vm.^38,40,41^ Bricchi et al.,^41^ studying the aphid *Myzus persicae* (Sulzer) (Hemiptera: Aphididae), observed a 12 Vm depolarization in response to feeding this aphid.

Plants have developed the ability to respond to herbivores, producing toxic compounds (such as secondary metabolites), new defense components,^4,42^ and molecular interactions that can attract predators or parasitoids of these herbivores.^43,43^ For example, GREEN and A,^44^ reported that tomatoes (*Lycopersicon esculentum*) respond to insect feeding by producing defensive proteins, such as proteinase inhibitors, that is, compounds that reduce protein digestion by insects and are induced in damaged leaves. Systemic transport signals are also involved when the translocation of defensive compounds contributes to systemic resistance, for example, when nicotine is produced in tobacco roots (*Nicotiana sylvestris*) when leaves are attacked.^45^

The electrophysiological signals from plants triggered by herbivore attack are complex and can lead to the activation of multiple defenses (Maffei et al., 2004),^55^ consequently causing morphological, physiological, biochemical, and molecular changes that affect plant growth and productivity. Therefore, determining which signaling pathways are involved in this regulation makes it possible to establish strategies to improve physiological performance and the development capacity and productivity of plants.^46^ In addition, the knowledge of stress-induced alterations in the membrane’s electrical potential and their effects allow the emergence of new stress monitoring tools, which is of paramount importance to elucidate the factors involved in these processes.

In recent studies, Pachu et al.^37,47^ characterized the production of electrical signals in Bt and non-Bt cotton plants (*Gossypium hirsutum* L.) infested with *Aphis gossypii* (Glover, 1877) (Hemiptera: Aphididae). The dispersal behavior of aphids was correlated with plant signaling responses. In their studies, the photosynthetic and electrical responses of the plant to the stress caused by the herbivory of *A. gossypii* combined with the stress generated using imidacloprid were evidenced.

The results obtained by PACHÚ et al.^37^ showed that both the Bt and non-Bt cotton varieties, when attacked by *A. gossypii*, emitted electrical signals of the variation potential kind and clearly showed the presence of distinct responses to the perception and behavior of aphids. Bt cotton plants propagated VP signals faster; however, they produced signals in a smaller amount with a higher density of aphids, further promoting greater dispersion of aphids within the plant. Despite this, there was a delay in terms of the number of signals propagated on Bt cotton plants with 60 aphids per plant, which produced the fewest number of signals between 0 and 36 h. Another important result was the greater dispersion behavior related to this same treatment, especially during and after 48 h of infestation.

Pachú et al.^37^ suggested that their results can be supported by two hypotheses and explained independently or combined. The first hypothesis is based on the possibility of a tradeoff in terms of defense of the Bt plant; a high dispersal could reflect a greater exploitation of food resources by aphids and facilitate the penetration of mouthparts by aphids on Bt cotton plants, which may explain why Bt cotton plants emitted faster electrical signals than non-Bt cotton plants in the former moment, showing that Bt cotton plants may be more susceptible to aphid stress. The second hypothesis is that the greater dispersal of aphids in Bt cotton may indicate that the first signals emitted by Bt cotton, even in smaller numbers than non-Bt cotton, were sufficient to activate the defense of that variety, preventing or making it difficult for aphids to feed.

Pachú et al.^47^ revealed that the application of imidacloprid in Bt and non-Bt cotton plants without the presence of the aphid led to variation potential (VP). These signals may have resulted in the inactivation or low efficiency of photosynthesis in some specific periods. Non-Bt cotton plants exposed to the insecticide and aphid resulted in low photosynthetic efficiency, indicating combined stress in this cultivar. The cotton respiration rate was also affected by insecticide application and aphid infestation. Bt cotton had a low respiration rate and low quantum yield, while non-Bt cotton had higher respiration and lower quantum yield.

## Electrical signals and activation of defence genes

According to Mudrilov et al.^9^ Electrical signaling is one of the most promising candidates for transmission of stimulus-specific re in the plants. Mousavi et al.^5^ observed that Arabidopsis thaliana plants, when attacked by Spodoptera littoralis larvae, generate electrical signals that were evoked at the damage site and spread to neighboring leaves.

In addition, Mousavi et al.^5^ found that the loss of function of certain members of the glutamate receptor family (GLR – GLUTAMATE RECEPTOR-LIKE) – some of which form channels permeable to calcium ions – affects injury-induced electrical signal generation. More specifically, the combined disruption of the genes that encode two of these channels, GLR3.3 and GLR3.6, results in the non-propagation of electrical waves after injury. Thus, tissue damage triggers the generation of a local electrical signal through the activity of GLRs; this signal then spreads to neighboring organs where jasmonic acid biosynthesis is induced and in turn triggers defense responses that are dependent on this hormone.

According to MEENA et al.,^36^ the high presence of calcium in plant cells is used by plants to recognize and signal environmental stress. The authors report CYCLIC NUCLEOTIDE GATED CHANNEL19 (CNGC19) as an activator of C2+ flow, which is induced by herbivory and plant defense. Vicente et al. (2017), points to the overactivation of Ca 2+ signaling as a potential mechanism to increase plant resistance to pests, considering the rapid and highly localized elevations of calcium in plants of the genus Arabidopsis after feeding by the green peach aphid *M. persicae*.

Indeed, previous studies have shown that mechanical damage alters the hydraulic pressure in the xylem, which in turn triggers a wave of depolarization that propagates through the plant (Stankovic & Davies, 1998). This mechanism of electrical signal generation is called the hydraulic hypothesis. According to this hypothesis, changes in xylem pressure caused by harmful stimuli (burning, cutting) trigger mechanosensitive channels present in the xylem parenchyma cells adjacent to the xylem vessels that trigger the generation of electrical signals, characterized as VP^27,48^. Stankovic and Davies (1998)^56^ also demonstrated that electrical signals induce proteinase inhibitor gene expression in tomato. Based on this information, it is possible to synthesize the activation of defense genes against herbivory.

According to our review presented we can conclude that plants have a complex system of perception and transmission of information about external environmental stimuli, which involves several different signaling systems and for a long time, electrical signaling was neglected at the expense of signaling studies in plants being concentrated with chemical and hydraulic signals. In recent years was revealed that plants are capable of emitting, processing, and transmitting bioelectrical signals to regulate a wide variety of physiological functions. In addition, many important biological and physiological phenomena are accompanied by these cellular electrical manifestations, which supports the hypothesis about the role of bioelectricity as a fundamental ‘model’ for response the stresses environmental and for activities regeneration of these organisms. Despite major advances in defining mechanisms involved in the generation and propagation of distance signals by stress, there are still a number of outstanding questions the effects of these signals have not been fully elucidated.
